# Potentiation of tumor responses to DNA damaging therapy by the selective ATR inhibitor VX-970

**DOI:** 10.18632/oncotarget.2158

**Published:** 2014-07-03

**Authors:** Amy B. Hall, Dave Newsome, Yuxin Wang, Diane M. Boucher, Brenda Eustace, Yong Gu, Brian Hare, Mac A. Johnson, Sean Milton, Cheryl E. Murphy, Darin Takemoto, Crystal Tolman, Mark Wood, Peter Charlton, Jean-Damien Charrier, Brinley Furey, Julian Golec, Philip M. Reaper, John R. Pollard

**Affiliations:** ^1^ Vertex Pharmaceuticals Inc, Boston, MA, USA; ^2^ Vertex Pharmaceuticals (Europe) Ltd, Milton Park, Abingdon, Oxfordshire

**Keywords:** ATR, DNA damage response, VX-970

## Abstract

Platinum-based DNA-damaging chemotherapy is standard-of-care for most patients with lung cancer but outcomes remain poor. This has been attributed, in part, to the highly effective repair network known as the DNA-damage response (DDR). ATR kinase is a critical regulator of this pathway, and its inhibition has been shown to sensitize some cancer, but not normal, cells *in vitro* to DNA damaging agents. However, there are limited *in vivo* proof-of-concept data for ATR inhibition. To address this we profiled VX-970, the first clinical ATR inhibitor, in a series of *in vitro* and *in vivo* lung cancer models and compared it with an inhibitor of the downstream kinase Chk1. VX-970 markedly sensitized a large proportion of a lung cancer cell line and primary tumor panel *in vitro* to multiple DNA damaging drugs with clear differences to Chk1 inhibition observed. I*n vivo* VX-970 blocked ATR activity in tumors and dramatically enhanced the efficacy of cisplatin across a panel of patient derived primary lung xenografts. The combination led to complete tumor growth inhibition in three cisplatin-insensitive models and durable tumor regression in a cisplatin-sensitive model. These data provide a strong rationale for the clinical evaluation of VX-970 in lung cancer patients.

## INTRODUCTION

DNA damaging agents such as cisplatin, etoposide, gemcitabine and ionizing radiation (IR) remain the cornerstone of cancer treatment, yet for most patients they provide only modest benefit. This has been attributed, in part, to the presence of a highly effective DNA damage surveillance and repair network[1-4]. A complex signaling pathway known as the DNA damage response (DDR) constitutes an important component of this network [5]. It is activated by some of the most lethal forms of DNA damage, including double strand breaks and replicative stress, which arise when DNA replication forks stall. Upon activation, the DDR triggers cell cycle checkpoints, cell survival pathways and DNA repair. The DDR is regulated by two homologous protein kinases, ataxia telangiectasia mutated (ATM), and ATM and Rad3-related (ATR)[6, 7]. ATM is activated by double strand breaks at all stages of the cell cycle. ATR, on the other hand, is recruited to tracts of single stranded DNA coated with replication protein A (RPA), a characteristic of stalled replication forks (replication stress) and resected double strand breaks that arise during the S and G2 phases of the cell cycle. While these two kinases respond to different DNA damage structures, they exhibit significant functional and signaling overlap. In keeping with this, inhibition of ATR can lead to activation of ATM and a compensatory DDR[8].

Despite the importance of the DDR for cell survival following DNA damage, defects in this repair pathway are common in cancer. For example, loss of ATM signaling capacity is frequently observed, either through loss of expression of ATM itself, or through defects in upstream regulators and downstream effectors such as p53[9-12]. It is believed that such defects in the DDR facilitate the emergence and proliferation of genetically unstable cancer cells[13]. Although defects in ATM signaling appear to confer a growth advantage, they cause a reliance on remaining DDR components, such as ATR, and thus provide an ‘Achilles’ heel' that could be targeted by new drugs. Consistent with this hypothesis we, and others, have shown that biological depletion or inhibition of ATR dramatically sensitizes cell lines lacking ATM or p53 function to DNA damaging drug mediated apoptotic cell death[8, 14-19]. In addition, mechanisms that increase replicative stress in cancer cells also appear to drive a reliance on the DDR, and ATR specifically, for survival. These include expression of common oncogenes such as Myc and Ras, and an hypoxic microenvironment[20-24].

Clinical evidence that DNA repair proficiency can influence response to DNA damaging therapy is emerging. For example, ovarian cancer patients harboring loss-of-function of the BRCA1/2 homologous recombination repair (HRR) proteins, respond better to platinum-based therapy than those patients with wild-type BRCA[4, 25, 26]. Furthermore, non-small cell lung cancer (NSCLC) patients with compromised ERCC1 function (a protein involved in various DNA repair pathways including HRR) have similarly improved responses to cisplatin-based therapy[2]. Taken together these observations have fueled interest in identifying drug candidates that target components of the DDR such as ATM, ATR, DNA-PK and the checkpoint kinases Chk1 and Chk2. Of these, only inhibitors of Chk1/2, and very recently of ATR, have entered clinical studies.

Lung cancer is the second most common cancer and a leading cause of cancer mortality. In the US alone it is estimated that over 200 000 new cases of lung cancer are diagnosed each year[27] [28]. Small cell lung cancer accounts for 10-15% of the overall lung cancer population with first line standard-of-care a doublet of a platinum drug (cisplatin or carboplatin) and etoposide. Although many patients initially respond to such treatment, almost all relapse[29]. NSCLC accounts for about 85% of the overall incidence of lung cancer[27]. The discovery of ‘targeted’ drugs such as erlotinib, that blocks aberrant EGFR signaling in patient with mutant EGFR, and crizotinib that blocks ALK signaling in patients expressing the EML4-ALK driver fusion protein, has led to improvements in NSCLC therapy for a small population of patients. However, the majority of patients are not candidates for treatment with such drugs[30] and for these patients standard-of-care remains platinum-based chemotherapy and survival is typically less than 12 months [28]. Clearly, there remains a high need for new therapies. Common characteristics of lung cancer, particularly NSCLC, are the widespread expression of oncogenes that drive replication stress (e.g. K-ras) and high frequency of defects in the ATM-p53 signaling pathway. For example, in one comprehensive analysis >80% of squamous cell NSCLC carried a mutation in the *TP53* gene, which is believed in many cases to lead to p53 loss-of-function[31]. This combination of high replication stress and defective ATM signaling may provide a strong reliance on ATR for survival following DNA damage. Consequently, the combination of an ATR inhibitor with agents such as cisplatin could be an attractive therapeutic option for lung cancer and for other indications that have a reliance on ATR for survival following treatment with DNA damaging therapy.

VX-970 (previously referred to as VE-822[18]) was the first potent and highly selective inhibitor of ATR to enter clinical studies (EUDRACT: 2012-003126-250, ClinicalTrials.gov: NCT02157792). In this report, extensive *in vitro* and *in vivo* evidence is provided to support the hypothesis that ATR inhibition can improve lung cancer patient responses to DNA damaging agents. Specifically, VX-970 markedly sensitized a large proportion of NSCLC cell lines, but not normal cells, to multiple DNA damaging drugs. Consistent with prior reports, matched cell lines differing only in p53 status confirmed that p53 loss of function, through deletion, influences cell sensitivity to ATR inhibition. In addition, VX-970 at well-tolerated doses, was shown to dramatically improve responses to cisplatin across a panel of primary patient-derived xenograft models. Finally, cell responses to VX-970 were compared with an inhibitor of the ATR substrate, Chk1. This highlighted significant differences between the two approaches, which may have important consequences to how these agents can be most effectively used in the clinic.

## RESULTS

### VX-970 inhibits cisplatin-induced phosphorylation of Chk1 and increases levels of DNA damage markers *in vitro*

**Figure 1:**
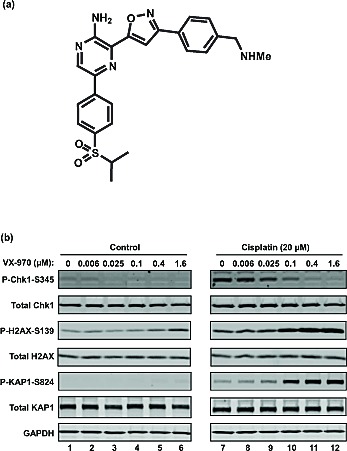
VX-970 is a potent and selective inhibitor of ATR (A) Chemical structure of VX-970. (B) Exponentially growing H2009 cells were treated overnight (17 h) with the indicated concentrations of VX-970 alone (*lanes 1-6*) or in combination with 20 μM cisplatin (*lanes 7-12*). Cells were then harvested and analyzed for the expression of P-Chk1-S345, P-H2AX-S139 and P-KAP1-S824 by immunoblotting.

VX-970 (also referred to as VE-822[18], Figure 1A) is a highly potent and selective, ATP competitive, inhibitor of ATR[18]. We previously reported that VX-970 inhibits ATR in biochemical assays with a Ki <200pM and that it has excellent selectivity over a panel of other kinases. Most notably VX-970 has >100-fold selectivity for ATR over the related phosphatidylinositol 3' kinase-related kinases (PIKK) including ATM and DNA-PK, both of which are involved in alternative DNA repair processes. We also showed that ATR potency and selectivity is retained in cells; VX-970 blocks ATR driven phosphorylation of H2AX with an IC50 of 19nM, compared with an IC50 of 2.6μM and 18μM for inhibition of ATM and DNA-PK mediated signaling respectively [18].

To demonstrate the impact of VX-970 on cisplatin-induced ATR signaling and DNA repair, H2009 lung cancer cells were treated with VX-970 and cisplatin as single agents or in combination (Figure 1B). ATR activity was assessed by measuring P-Chk1 (S345) and DNA damage accumulation by P-H2AX (S139) and P-KAP1 (S824). VX-970 treatment alone inhibited background levels of P-Chk1 and led to mild induction of P-H2AX and P-KAP1. Cisplatin alone resulted in elevation of P-Chk1, P-H2AX and P-KAP1 above background. Co-treatment with VX-970 and cisplatin resulted in a VX-970 concentration-dependent suppression of cisplatin-induced P-Chk1 and a concurrent marked increase in P-H2AX and P-KAP1. These data are consistent with inhibition of ATR leading to the accumulation of DNA damage.

### VX-970 synergizes with DNA damaging agents in lung cancer cell lines

**Figure 2:**
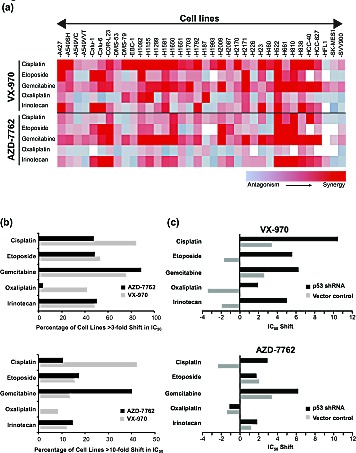
ATR inhibition sensitizes lung cancer cells to DNA damaging agents (A) Analysis of shifts in the concentration of DNA damaging agent required to inhibit cell viability by 50% (IC_50_) was used to determine the synergistic or antagonistic effects of a DDR inhibitor. Heat map representing the IC_50_ shift with the largest absolute value observed with VX-970 or AZD7762 in combination with cisplatin, etoposide, gemcitabine, oxaliplatin and irinotecan across a panel of 36 lung cell lines at 96 h. The colors represent a shift range from −10 (antagonism-blue) to +10 (synergy-red). (B) Histograms showing the percentage of cell lines with > 3-fold (top panel) or 10-fold (bottom panel) synergy with VX-970 (ATR) or AZD7762 (Chk1/2) in combination with cisplatin, oxaliplatin, irinotecan, gemcitabine and etoposide. (C) Impact of p53 on response to VX-970 in A549 cells. Histogram depicts maximum IC_50_ shift in vector control and p53 knockdown cells observed with VX-970 in combination with cisplatin, etoposide, gemcitabine, oxaliplatin and irinotecan.

Inhibition of ATR or its downstream substrate, Chk1, has been shown to sensitize cancer cells to certain DNA damaging drugs and ionizing radiation (IR). The long-held view has been that these two proteins operate on the same linear pathway[32]. However, a recent study using VE-821 (an analog of VX-970) and a Chk1 inhibitor, in a small number of ovarian cancer lines, concluded that the sensitization profiles of these two compounds can be different[33]. In order to get a better understanding, we assessed the ability of VX-970 and the Chk1 inhibitor AZD7762[34] to sensitize a panel of 35 lung cancer cell lines to five DNA damaging drugs: cisplatin, oxaliplatin, gemcitabine, etoposide and SN38, the active metabolite of irinotecan (Figure 2, Supplementary Figures 1 and 2, Supplementary Table 1). Sensitization was determined by measuring the maximum shifts in IC_50_ values (cell viability) for the DNA damaging drug in the presence or absence of the DDR inhibitor. The data are presented as a heatmap (Figure 2A) and as histograms that show the percentage of lines with >3-fold or >10-fold shifts in IC_50_ (Figure 2B). VX-970 sensitized (defined by >3-fold IC_50_ shift) over 40% of cell lines to all of the five drugs. The effect of VX-970 was most evident with cisplatin or gemcitabine co-treatment, where >75% of cell lines were sensitized (>3-fold IC_50_ shift). In contrast, against a non-cancer lung cell line (HFL1), sensitization was only observed in combination with gemcitabine (Figure 2A). AZD7762 showed a similar profile to VX-970 in combination with gemcitabine, SN38 and etoposide. However, in combination with platinating drugs, AZD7762 was far less effective than VX-970 (<50% cells sensitized to cisplatin and <10% sensitized to oxaliplatin). When the data were analyzed to identify highly responsive lines (defined by >10-fold IC_50_ shift), we found that VX-970 hypersensitized >40% of cell lines to cisplatin. In contrast AZD7762 hypersensitized only 10% of cell lines to cisplatin, but was more effective in combination with gemcitabine than VX-970 (40% of lines hypersensitized to AZD7762 vs 14% with VX-970). These data show that ATR and Chk1 inhibitors have different sensitization profiles, with VX-970, the ATR inhibitor, most effective in combination with platinating agents and AZD7762, the Chk1 inhibitor, most effective in combination with gemcitabine.

**Table 1:** VX-970 sensitizes primary human lung tumor cells to cisplatin *in vitro*

Tumor ID	MAX IC_50_ Shift	Cisplatin IC_50_ (μM)
OD35982	0.9	3.0
OD29498	1.2	3.2
OD26749	1.3	5.1
OD26131	2.1	16.8
OD33966	4.2	5.2
BDG121410	43.0	29.1
OD29607	1112.2	14.9

IC_50_ values for cisplatin calculated from *in vitro* human primary tumor experiments and the maximum shift in IC_50_ value for cisplatin in the presence of VX-970.

Using isogenically matched cell pairs, a number of studies have demonstrated that disruption of ATM/p53 function promotes sensitivity to inhibition of the ATR pathway. This has been attributed to a synthetic lethal relationship between the ATR and ATM signaling pathways[8, 14-17, 19]. However, it has also been shown that ATR pathway inhibitors can still sensitise ATM/p53 competent cells to DNA damaging agents[8, 14, 21, 35]. To examine the impact of p53 status on cellular response, we first assessed VX-970 in combination with five DNA damaging drugs on a pair of isogenic A549 cells stably transfected with either p53 shRNA or a scrambled RNA control vector. In all cases the combination of VX-970 with a DNA damaging drug was more effective in the p53 knockdown line (Figure 2C). In contrast, responses for AZD7762 where only affected by p53 function in combination with gemcitabine and cisplatin. To investigate the role of p53 across a heterogeneous set of cell lines we looked at the correlation of *TP53* mutational status with cell sensitivity (defined by >3-fold IC_50_ shift). *TP53* mutational status was used as this is readily measurable in the clinic, in contrast to p53 function. Although no significant correlations were observed (*P*>0.05), an interesting but non-significant trend was found with the combination of VX-970 and cisplatin (odds ratio of 6.1, *P* <0.08). For all other combinations no correlation was observed. Furthermore, no correlation was found with response and histological background of the cell lines (data not shown).

### VX-970 synergizes with cisplatin in primary human lung tumor cells *in vitro*

**Figure 3:**
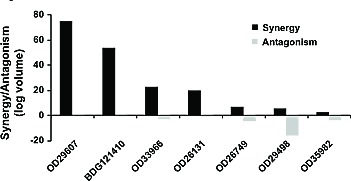
VX-970 synergizes with cisplatin across a range of human primary lung tumor models *in vitro* Dissociated tumor cells were treated in triplicate *in vitro* with a matrix of VX-970 and cisplatin concentrations, and synergy or antagonism was analyzed at the 95% confidence interval with MacSynergy II software. Degree of synergy, shown as positive log volume, and antagonism, shown as negative log volume, are shown.

To further explore the clinical potential of VX-970, we assessed it in combination with cisplatin *in vitro* against seven primary human NSCLC tumors from a range of histopathological subtypes (Supplementary Table 3). Responses were determined by CellTiter-Glo as a measure of cell viability and synergy was assessed using a statistical Bliss analysis (Figure 3) or by determining the impact of VX-970 on the IC_50_ for cisplatin (Table 1). The seven tumors showed varying responses to cisplatin monotherapy with IC_50_ values over the range 3.0 μM – 29.1 μM. Marked synergy (log volume >20) was observed between VX-970 and cisplatin for four of the seven tumors, and for three of these the IC_50_ for cisplatin was reduced by >3-fold on addition of VX-970. Interestingly, tumors with poor response to cisplatin monotherapy were the tumors that demonstrated the greatest synergy with VX-970. There was no apparent relationship between *TP53* status and response. Consistent with observations on the H2009 cell line, treatment of these primary tumors with VX-970 led to a concentration-dependent inhibition of cisplatin-induced P-Chk1 and elevation of P-H2AX and P-KAP1 (Supplementary Figure 3).

### VX-970 enhances the efficacy of cisplatin in patient-derived lung tumor xenografts

**Figure 4:**
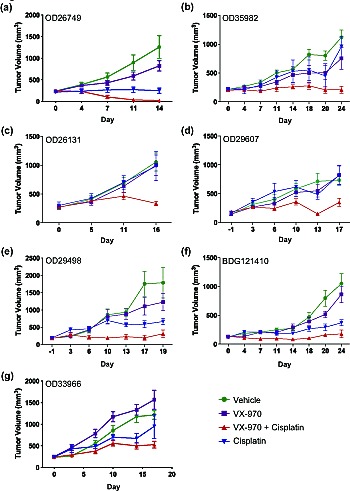
VX-970 enhances the therapeutic efficacy of cisplatin in patient-derived lung tumor xenografts (A-G) Human primary tumor tissues were passaged in SCID mice. Treatment started when the average tumor size was approximately 200 mm^3^. Tumor bearing mice were treated with vehicle, VX-970 (30 mg/kg in all models except 60 mg/kg in OD26749 and OD26131) PO, 4 consecutive days a week, alone and in combination with cisplatin (3 mg/kg IP, q7d), and cisplatin alone. Tumor volume and body weight were measured twice a week. Studies were terminated one or two days after the final dose of VX-970. Points show the mean tumor volume (mm^3^) for each treatment group (n=5-10).

We next tested the efficacy of VX-970 in combination with cisplatin in SCID mice implanted with each of the seven primary lung tumors (Supplementary Table 3). Oral doses of VX-970 at 30 or 60 mg/kg for four consecutive days each week, alone or in combination with weekly IP doses of cisplatin at 3 mg/kg were well tolerated with a non-statistically significant decrease in body weight in some of the combination groups immediately following initiation of treatment (Supplementary Table 4). Body weight was gained between treatment cycles (a typical body weight profile is provided in Supplementary Figure 4 for tumor OD35982). VX-970 alone showed no significant single agent effect in any of the seven models. Cisplatin, on the other hand, gave a range of responses: three tumors were sensitive to cisplatin monotherapy (tumor growth inhibition >70%; OD26749, OD29498, BDG121410), one tumor was weakly sensitive (tumor growth inhibition 50-70%; OD33966), and three were resistant to cisplatin (tumor growth inhibition <20%; OD35982, OD26131, OD29607), Figure 4). Addition of VX-970 led to a statistically significant enhancement of cisplatin efficacy in six of the seven models examined (*P* ≤ 0.05; for BDG121410 P=0.1). Notably, the combination led to complete tumor growth inhibition in the three-cisplatin insensitive models and complete tumor regression in one cisplatin sensitive model (OD26749 model, Figure 4A). We selected the OD26749 model to further characterize the response of primary lung xenograft tumors to VX-970 alone and in combination with cisplatin.

### VX-970 inhibits P-Chk1 and causes the accumulation of DNA damage in primary human xenografts

**Figure 5:**
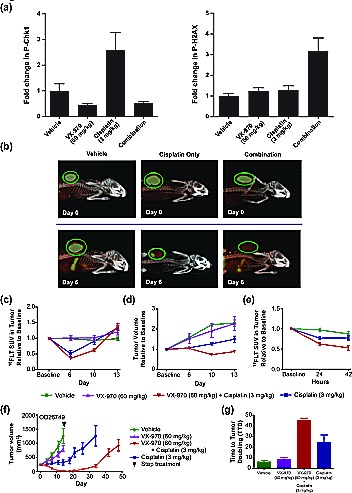
VX-970 inhibits ATR and promotes sustained regression in a human primary lung tumor xenograft (A) Mice bearing OD26749 tumors were treated with a single dose of VX-970 (60 mg/kg PO) and cisplatin (3 mg/kg IP) either as monotherapy or in combination. P-Chk1 and P-H2AX were analyzed by western blot 4 and 48 h after treatment, respectively, and were corrected to total H2AX levels. (B) Representative PET/CT images of mice at baseline (top row) and six days following the indicated treatment (bottom row). Concentration of ^18^FLT is indicated by the intensity of the orange color in the PET image. An X-ray CT image of the skeletal system (white) is superimposed for anatomical reference. The tumor is circled (green). Treatment with VX-970 alone had no discernable effect on PET signal (image not shown). (C) Average standard uptake value (SUV) of ^18^FLT. (D) Tumor size changes as determined by CT. (E) Examination of prognostic value of ^18^FLT imaging. (F) Assessment of the durability of response to VX-970. OD26749 tumors were passaged in SCID mice to P4. Treatment started when the average tumor size was approximately 200 mm^3^. Tumor bearing mice were treated with vehicle, VX-970 alone (60 mg/kg PO, 4 consecutive days a week), cisplatin alone (3 mg/kg, IP, q7d) and the combination, for two weeks. Vehicle and VX-970 groups were terminated after the final dose of VX-970. Following treatment discontinuation, tumor growth in the cisplatin alone and combination groups was followed twice a week until the average tumor volume reached 1000 mm^3^. The arrowhead on the graph marks the end of treatment. Error bars are standard errors for all graphs.

Mice bearing OD26749 tumors were given a single dose of VX-970 (60 mg/kg PO) and cisplatin (3 mg/kg IP) either as monotherapy or in combination. ATR activity was measured by P-Chk1 western blot four hours after treatment (coincident with the C_max_ for VX-970) and DNA damage was measured by P-H2AX western blot, 48 hours after treatment (Figure 5A, Supplementary Figure 5). Treatment with VX-970 led to a significant inhibition of cisplatin-induced P-Chk1 and elevation of P-H2AX (*P* ≤ 0.05). In a separate study, tumor concentrations of VX-970 were determined from a single oral dose of 60 mg/kg. Correcting for protein binding, the maximum free–drug tumor concentration (Cmax) of VX-970 was 720nM, 4 hours after dosing. During the 48-hour period following administration of VX-970, free-drug tumor concentrations were maintained at over 230nM. These concentrations are substantially in excess of the cell IC_50_ for inhibition of ATR (19nM) but below the cell IC_50_ for inhibition of ATM or DNA-PK (2.6μM and 18μM respectively). Together the results indicate that at doses used in this study (60mg/kg), VX-970 blocks ATR (but not ATM or DNA-PK) activity in tumors, leading to disruption of DNA repair and accumulation of cisplatin-mediated DNA damage.

### ^18^F-fluorodeoxythymidine (^18^FLT) positron emission tomography (PET) is an early marker of tumor response to VX-970

^18^FLT, an analog of the DNA nucleotide thymidine, is taken up by proliferating cells and serves as a non-invasive imaging tracer of proliferative index, which can be detected by PET[36]. Mice bearing OD26749 tumors were treated with single doses of VX-970 (60 mg/kg PO) and cisplatin (3 mg/kg IP) individually or in combination. ^18^FLT uptake was assessed over a period of 13 days. Untreated tumors showed a pronounced uptake of ^18^FLT, with the standardized uptake value (SUV) greatly exceeding that seen in surrounding normal tissues (Figure 5B and C). Six days following treatment with cisplatin alone, the tumor SUV had dropped by nearly half compared to vehicle control (*P* <0.01, Figure 5B and C). In contrast, administration with VX-970 alone at the doses used in this study had no effect on tumor SUV. Co-administration with VX-970 and cisplatin caused a mean drop in SUV that was greater than with cisplatin treatment alone (*P*<0.05 at day 10; Figure 5C). Tumor size, as measured by CT (Figure 5D), correlated with probe uptake. Co-administration of VX-970 with cisplatin led to significantly greater inhibition of tumor growth than was observed with cisplatin or VX-970 alone, (*P* <0.01 at Day 10). To assess whether ^18^FLT uptake could be used as a very early marker for tumor response, we examined tracer uptake during the 42 hours following initiation of treatment (Figure 5E). By 24 hours after treatment, suppression of ^18^FLT uptake in the combination treated mice was significantly greater than in mice treated with cisplatin alone (38 % vs. 24 % respectively, *P* <0.05) and this difference was even more apparent by 42 hours (47 % vs. 23 %, *P* = 0.01). These data support the potential for ^18^FLT uptake as a prognostic biomarker of response to VX-970 in combination with DNA damaging drugs such as cisplatin.

### VX-970 and cisplatin combinations cause significant regression and delayed tumor regrowth in a human primary tumor xenograft model

The durability of response to VX-970 with cisplatin was assessed in mice bearing OD26749 tumors. Mice were treated for two weeks with VX-970 daily at 60 mg/kg PO for four consecutive days each week and with cisplatin at 3 mg/kg IP once weekly. The combination induced profound and near complete tumor regression (Figure 5F) whereas cisplatin treatment alone only induced modest tumor growth inhibition. Upon cessation of treatment with the combination of VX-970 and cisplatin, tumor growth remained suppressed for three weeks before tumors started to grow back. The time-to-tumor-doubling from the start of the study (TTD) for the combination was 45 days, significantly longer than for cisplatin alone (25 days *P* <0.05).

## DISCUSSION

Double strand DNA breaks and replicative stress can lead to loss of genomic integrity and cell death. Consequently, cells have evolved a comprehensive surveillance and repair mechanism termed the DDR[5]. Growing evidence suggests that an effective DDR is critical for cancer cell survival following treatment with many DNA damaging anti-cancer drugs. Therefore much effort has been expended on the discovery and development of inhibitors of the DDR that could be used to enhance the efficacy of DNA damaging agents[37, 38]. Until recently, Chk1-targeted agents have been the only inhibitors of DDR signaling to progress into the clinic. However, more recently, two ATR-targeted drug candidates have been reported (VX-970, also referred to as VE-822[18], and AZD6738). In previous reports we have shown that VX-970 is a highly potent inhibitor of ATR in biochemical (ATR Ki <0.2nM) and cell based assays (ATR IC_50_ 19nM) with excellent selectivity over the related kinases ATM and DNA-PK and a panel of unrelated kinases[18]. This study describes the impact of inhibiting ATR by VX-970 on cell and tumor responses to DNA damaging drugs.

VX-970 blocks cisplatin induced P-Chk1 (S345) and elevates the levels of two DNA damage markers (P-H2AX at S139 and P-KAP1 at S824) in a concentration-dependent manner in cancer cell lines and primary human tumors. These changes are evident with concentrations of VX-970 as low as 100nM, significantly below the concentrations required to impact the activity of the other DNA repair kinases ATM and DNA-PK (IC_50_ values of 2.6μM and 18μM respectively[18]). This is consistent with the notion that inhibition of ATR impairs repair of DNA lesions leading to accumulation of damage[6, 39]. Phosphorylation of Chk1, H2AX and KAP1 could serve as clinical pharmacodynamic markers for ATR-targeted drug-candidates. However, in a recent study [33], VE-821 (a close analog of VX-970) failed to impact P-Chk1 (Ser-345) in ovarian cancer cell lines despite causing an elevation of P-H2AX. Therefore, we cannot discount the possibility that other kinases are able to phosphorylate Chk1 at Ser345 in certain contexts. To address this possibility, a comprehensive analysis of ATR-mediated phosphorylation of Chk1, across different cell types and during extended time courses, is warranted.

Several studies have used small numbers of cell lines to assess the potential for inhibition of the DDR to sensitize cancer cells to various DNA damaging agents. From these studies a couple of interesting observations emerged. First, despite ATR and Chk1 acting on the same signaling cascade[32], inhibition of these individual proteins can have quite different consequences [33, 40]. Second, disruption of p53 function can influence cellular response to inhibition of the DDR[8, 14-17, 19, 41-43]. This has been attributed to a synthetic lethal relationship between ATR and the ATM-p53 signaling pathway[8]. We sought to elaborate on these two observations by assessing the effects of the ATR inhibitor, VX-970, and the Chk1 inhibitor, AZD7762, on an extensive panel of lung cancer cell lines in combination with five DNA damaging drugs from various mechanistic classes. VX-970 sensitized many of the cell lines to all five DNA damaging agents. It sensitized cells to cisplatin most effectively with over 80% of lines responding. Of these, half exhibited greater than 10-fold increases in sensitivity to the DNA damaging agent. In addition, in a small study VX-970 was shown to combine very effectively with carboplatin in two lung cancer cell lines (>10-fold shift in carboplatin IC_50_, data not shown). The remarkable response to cisplatin is consistent with recent data implicating ATR in the activation of the Fanconi Anaemia DNA crosslink repair pathway, defects in which are characterized by hypersensitivity to interstrand crosslinking agents[44-46]. Taken with its established roles in the DDR, this may explain why inhibition of ATR is so effective in combination with cisplatin. The combination of VX-970 with oxaliplatin, another platinum based cross-linking drug, was however generally less effective than with cisplatin. These data suggest that subtle differences in the structure of the cross-link lesion may lead to different damage response pathways being activated.

A comparison of the overall profiles for AZD7762 and VX-970 highlights some interesting differences. Although AZD7762 sensitized some cancer cells to cisplatin, it was considerably less effective than VX-970. This decreased cell sensitivity to AZD7762 compared with VX-970 extended to combinations with oxaliplatin. On the other hand, although VX-970 was effective in combination with gemcitabine in most cells, AZD7762 appeared more effective. This was most evident from the higher proportion of cell lines that were hypersensitized (>10-fold shifts in gemcitabine IC50) to gemcitabine by AZD7762 *vs* VX-970. Taken together, these findings support ATR-dependent but Chk-independent survival responses to certain DNA cross-linking agents. In addition, our data indicate that AZD7762 may be a more effective mechanism to sensitize cells to gemcitabine than a selective ATR inhibitor like VX-970. This may be attributable to the fact that AZD7762 also has Chk2 inhibitory activity[34]. Clearly, more work is required to fully elucidate the impact of different replication stress structures on DDR signaling. To address the role of p53 in determining cell fate following co-administration with inhibitors of the DDR and DNA damaging drugs, we studied the impact of p53 knockdown using an isogenically controlled cell pair and also analyzed the full cell panel for statistical correlations between cell response and *TP53* status (since *TP53* status can be readily assessed in the clinic in contrast to p53 function). Consistent with previous reports using isogeneic cell systems[8, 14-17, 19], we showed that p53 loss increased cell sensitivity to ATR inhibition in combination with all five DNA damaging drugs. However, in the context of an extensive and heterogeneous cell panel, using *TP53* status, the picture became less clear. Only for the combination of VX-970 and cisplatin was a relationship observed between response and *TP53* status, however this did not reach statistical significance (*P* =0.08). Furthermore, when VX-970 was tested in combination with cisplatin in a panel of seven primary lung tumors, there was no clear relationship between cell response to VX-970 and *TP53* status. A number of hypotheses can be provided to explain the differences between isogenic cell responses and the large cell panel data. Firstly, not all *TP53* mutations lead to loss of p53 function. Conversely some *TP53* wild type cells may be sensitive to ATR inhibition if there are defects elsewhere in the ATM-p53 pathway. This situation has been reported with the HCT116 colorectal cancer cell line that is highly sensitive to ATR inhibitors despite being wild type for *TP53*. In this case, defects in both ATM expression and activation have been described[8]. Additionally, high background levels of replication stress would also place a significant reliance on the ATR repair pathway, which may be independent of ATM pathway function. For example, it has been shown that expression of oncogenes such as Myc, Ras or Cyclin E, which cause disregulated DNA replication, drive high levels of replicative stress[13]. Cells expressing such oncogenes have been reported to be highly sensitive to ATR inhibition or depletion, in the absence of additional DNA damaging treatment. In all cases these cells expressed wild type *TP53*[14, 20-22]. Predicting which tumors will respond to treatment with drugs like VX-970 is clearly of great clinical relevance and further work is warranted.

To assess the chances of success in the clinic, we looked at VX-970 in combination with cisplatin in a panel of seven xenograft models derived from primary human NSCLC samples. At doses where VX-970 had no effect as a monotherapy, it significantly improved responses to cisplatin (*P* ≤0.05) in six out of the seven models. Notably, three of the seven tumors were not responsive to cisplatin monotherapy, however co-treatment with VX-970 caused complete tumor growth inhibition. Additionally, in one cisplatin sensitive model (OD26749) the combination of VX-970 and cisplatin led to rapid, complete and durable regression. In all *in vivo* studies the combination of cisplatin and VX-970 was well tolerated. Free drug tumor levels of VX-970 were maintained for 48 hours after administration at concentrations well over that required to block ATR activity in cells. However, even at Cmax, the free drug concentrations of VX-970 were significantly below that required to block ATM or DNA-PK activity in cells. This, coupled with biomarker data showing blockade of ATR activity and accumulation of DNA damage is consistent with the anti-cancer activity of VX-970 being driven by inhibition of ATR and subsequent impaired DNA damage repair.

Finally, we assessed the potential for PET imaging, using the ^18^FLT tracer, as an early marker of tumor response to co-treatment with VX-970 and cisplatin. Treatment with cisplatin alone resulted in a marked decrease in ^18^FLT uptake. The addition of VX-970 to cisplatin led to a further decrease in ^18^FLT uptake at nadir and also altered the kinetics of uptake; decreased ^18^FLT uptake occurred earlier and was sustained for longer in the combination group than with cisplatin alone. The increased impact on ^18^FLT uptake for the combination group *vs* cisplatin monotherapy was reflected in improved tumor growth inhibition. Importantly, differences in ^18^FLT uptake between the combination and cisplatin monotherapy groups were discernible before differences in tumor size could be detected. This supports the use of ^18^FLT-PET imaging as an early marker of efficacy in the clinic.

In the studies described here, we have used a range of translational *in vitro* and *in vivo* models to demonstrate the potential VX-970 may have to increase the efficacy of DNA damaging therapy in patients with lung cancer. Our results support a proposal that inhibition of ATR will provide a new approach to improve patient responses to the DNA damaging agents that form the current standard-of-care across many cancer indications.

## METHODS

### Compounds

SN-38 (active metabolite of irinotecan), etoposide, cisplatin and oxaliplatin were obtained from Sigma-Aldrich, gemcitabine from Sequoia Research Products and AZD7762 from Thermo Fisher.

### Cell Lines

COR-L23 and EBC-1 cell lines were obtained from the European Collection of Cell Lines (ECACC) and the Japanese Collection of Research Bioresources (JCRB), respectively. All other cell lines were purchased from American Type Culture Collection (ATCC). No additional in-house authentication of cell lines was carried out. Cells were cultured in DMEM high glucose (Life Technologies) supplemented with 10% heat-inactivated FBS (Hyclone).

### Immunoblotting Analysis

Extracts were prepared in SDS-PAGE sample buffer and immunoblotted as described previously[8] using antibodies for Chk1 (Santa Cruz), phospho-Chk1-S345 and H2AX (Cell Signaling), phospho-H2AX-S139 (Millipore), phospho-KAP1-S824 (AbCam), KAP1 (Thermo Fisher) and GAPDH (Life Technologies).

### Cell Line Viability and IC_50_ Analysis

Compounds were added in a 5-concentration dose response for DDR inhibitors (VX-970: 25 nM-2 μM and AZD7762: 62 nM-5 μM) and a 6-concentration dose response for genotoxic agents (etoposide: 10 nM-10 μM, gemcitabine: 0.16 nM-160 nM, cisplatin: 20 nM-20 μM, oxaliplatin: 40 nM-40 μM, SN-38: 0.12 nM-120 nM). Cells were incubated for 96 h and viability was measured using CellTiterGlo (Promega). Genotoxin IC_50_ values were calculated using DMSO-normalized cell survival values. Genotoxin IC_50_ values in the presence of DDR inhibitor were calculated using DDR inhibitor-normalized cell survival values. IC_50_ values were calculated using the R package drc (University of Copenhagen). Statistical analyses were also run using the R package[47]. Maximum IC_50_ shifts were calculated using the maximum absolute value of the ratio of the genotoxic IC_50_ at a given DDR inhibitor concentration to the IC_50_ with no DDR inhibitor. Heat maps were generated using the Spotfire software package (TIBCO Software, Inc)

### Primary Tumor *In Vitro* Chemosensitivity Assay

Human tumor xenografts were resected from mice and the cells were dissociated as described previously[48]. Dissociated cells were washed and resuspended in PC-1 medium (Invitrogen) and plated in ultra-low attachment plates (Costar). Cisplatin was tested from 70 nM to 50 M and VX-970 from 4 nM to 3 μM. Cell viability was measured after 6 days using the CellTiter-Glo assay (Promega). Synergy and antagonism were assessed according to the Bliss independence model as described previously (8).

### Animals

Female Severe Combined Immunodeficiency (Fox Chase SCID, CB-17) mice weighing 17-19 g (Charles River Laboratories) were acclimatized with access to food and water ad libitum and handled in accordance with IACUC regulations and guidelines.

### Ethics Statement

Investigation has been conducted in accordance with the ethical standards and according to the Declaration of Helsinki and according to national and international guidelines and has been approved by the authors' institutional review board.

### Xenograft Studies

Human lung tumor samples were procured by the National Disease Research Interchange (NDRI) and Tissue Solutions Ltd. Tumor xenograft models were established by implanting fresh patient tumor fragments into SCID mice. The tumor line was expanded and maintained by serial passage of tumor fragments (50-150 mg) subcutaneously (SC). To promote growth, 75 to 100 μL of Matrigel (BD Biosciences) supplemented with growth factors was injected SC into the site of implantation. VX-970 was dissolved in 10% Vitamin E d-alpha tocopheryl polyethylene glycol 1000 succinate (TPGS) and administered by oral gavage at 30 mg/kg for all primary tumor models except OD26749 and OD26131 (60 mg/kg) in a dosing volume of 10 mL/kg. Cisplatin (Sigma) was solubilized in injectable saline (0.9 % sodium chloride, ThermoFisher) and administered by intraperitoneal (IP) injection at 3 mg/kg in a dosing volume of 10 mL/kg. Randomization was initiated when the average tumor size was approximately 200 mm3. Tumor bearing mice were administered with VX-970 (PO, 4 consecutive days a week; OD26749, OD26131, OD35982, OD29607, OD29498, BDG121410 and OD33966) alone and in combination with cisplatin (3 mg/kg IP, q7d). Depending on the rate of tumor growth, two (OD26749 and OD26131) or three treatment (OD35982, OD29607, OD29498, BDG121410 and OD33966) cycles were performed during the course of the study. For the OD26749 recurrence study, two treatment cycles were performed and then tumor growth was monitored until the mean tumor volume reached 1000 mm3. Tumor volumes and body weights were measured twice a week starting on the first day of treatment until study termination. Tumor volumes were calculated using the formula (length x width2)/2. Fold difference in final mean tumor volume for the cisplatin alone and combination treatment groups was determined. For the OD26749 recurrence study, time-to-tumor doubling (TTD) was reported as the time required for the average tumor volume to increase by 2-fold compared with the average starting volume.

### Positron Emission Tomography (PET)

Animals bearing OD26749 xenografts were randomized into vehicle, cisplatin only (3 mg/kg IP), VX-970 only (60 mg/kg PO) and a combination group. The dosing regimen for the 13-day study (Figure 5, B-D) consisted of a single dose of VX-970 and cisplatin. A subsequent experiment examining early response (Figure 5E) consisted of three treatment groups: vehicle, cisplatin only (3 mg/kg IP single dose), and combination of cisplatin and VX-970 (60 mg/kg VX-970 PO). All animals received an intravenous injection of ^18^F-FLT (PETNET Solutions) at 5 μCi/g in the lateral tail vein. After one-hour uptake, mice were anesthetized using 2 % isofluane (Baxter) and positioned prone in a four-mouse bed. X-Ray Computed Tomography images were acquired using an Inveon Multimodality CT (Siemens Preclinical Solutions) and reconstructed using a cone beam algorithm into 384 × 384 × 602 image arrays with a 217 μm pixel size. The bed was then advanced into a mechanically-docked Inveon PET scanner (Siemens Preclinical Solutions) without mice being removed from the bed. PET emission data was acquired for 15 minutes. Images were reconstructed using a 2D Filtered Back Projection algorithm corrected for scatter into 128 × 128 × 159 image arrays with a 0.77 mm pixel size and 0.796 mm slice thickness. CT images were spatially aligned to the PET images using a predefined fixed transformation matrix and used for attenuation correction of the PET scans. Processing of reconstructed images was performed using Inveon Research Workplace (Siemens Preclinical Solutions). The mouse xenograft tumor was defined on the CT image without the reference to the PET overlay image. 3D regions of interest were created from interpolated 2D regions of interest drawn every eight slices in the axial view. Volume of the xenograft was obtained from the CT image. PET voxel intensity values were converted to standard uptake values (SUV) by the usual equation: SUV = activity in region of interest (ROI) / (injected dose / mouse body weight), after accounting for decay of injected isotope.

### Statistical Analysis

Odds ratio and *P* values for *TP53* mutational analyses were calculated using one-sided Fisher test. Unpaired, two tailed t-tests on tumor volume were used to evaluate statistical significance between treatment groups in efficacy studies using human primary models.

## SUPPLEMENTARY MATERIAL FIGURES AND TABLES


