# Supplementary material for: Potentiation of tumor responses to DNA damaging therapy by the selective ATR inhibitor VX-970
Source: Oncotarget. 2014 Jul 3;5(14):5674–85. doi: 10.18632/oncotarget.2158 (PMC4170644; doi:10.18632/oncotarget.2158)
Supplement: Supplementary file 1 [file oncotarget-05-5674-s001.pdf]

Potentiation of tumor responses to DNA damaging therapy by the selective ATR inhibitor VX-970

Supplementary Material

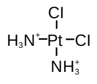

Cisplatin

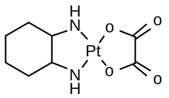

Oxalipatin

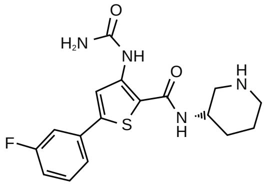

AZD-7762

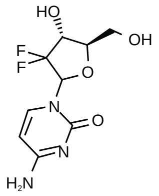

Gemcitabine

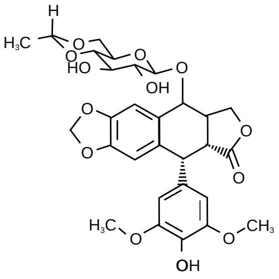

Etoposide

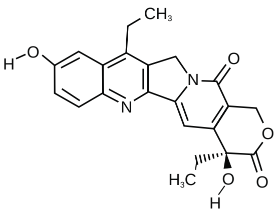

Irinotecan

Supplementary Figure 1: Chemical structures of AZD7762 (Chk1/2), oxaliplatin, cisplatin, irinotecan, gemcitabine and etoposide.

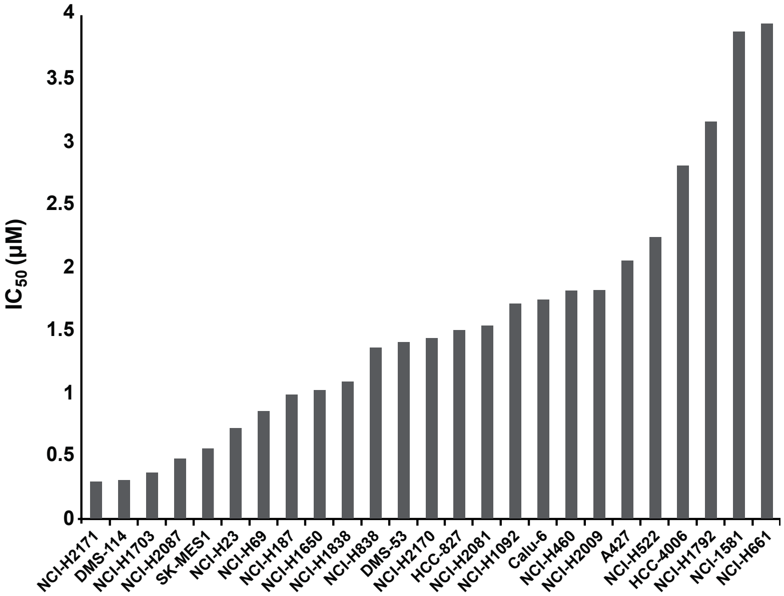

Supplementary Figure 2: Single agent activity of VX-970 against a panel of lung cell lines. IC_{50} values of single agent VX-970 across a panel of lung cell lines determined using CellTiterGlo

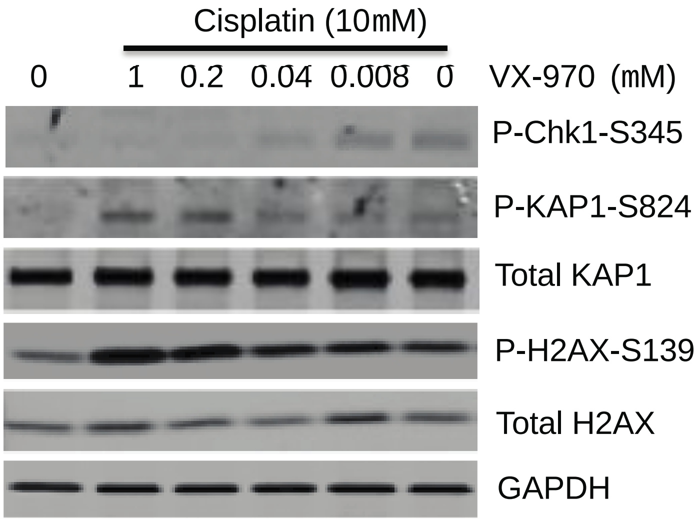

Supplementary Figure 3: Impact of VX-970 and cisplatin treatment on markers of ATR activity and DNA damage in primary lung tumor cells. Cells dissociated from tumor OD26749 were allowed to grow for 3 days and exposed in vitro to VX-970 (0, 0.008, 0.04, 0.2, 1 µM) plus cisplatin (10 µM). Extracts were prepared after 8 h of treatment and immunoblotted as indicated. GAPDH, glyceraldehyde 3-phosphate dehydrogenase.

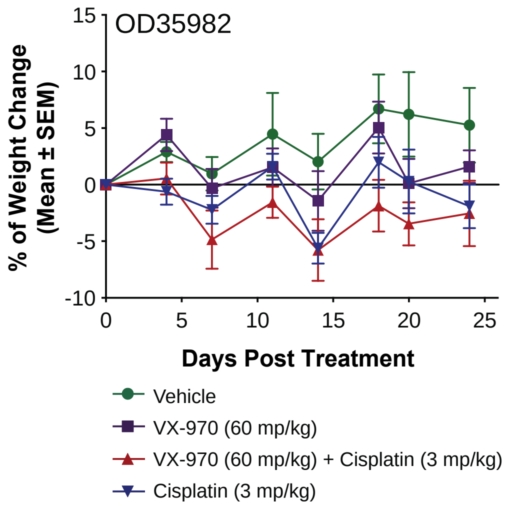

Supplementary Figure 4: Effect of VX-970 and / or cisplatin on body weight in the OD35982 primary lung tumor xenograft model. Mice bearing OD35982 tumors were treated with vehicle, VX-970 alone (30 mg/kg PO, 4 consecutive days a week), cisplatin alone (3 mg/kg, IP, q7d) and the combination, for three weeks. Cisplatin was administered 16 hours following the first dose of VX-970 of each cycle on Day 17, Day 24 and Day 31 after tumor implantation. Treatment was stopped on Day 34 post implantation. Mouse body weights were measured twice a week.

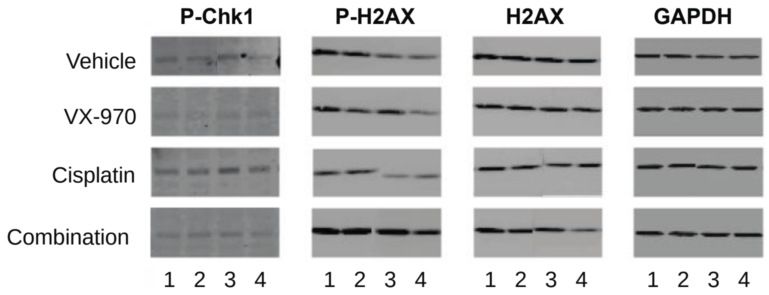

Supplementary Figure 5: Western blots of markers of ATR activity and DNA damage from primary lung tumors taken from mice treated with VX-970 and / or cisplatin. OD26749 tumors were harvested from four mice (1-4) dosed once with Vehicle, 60 mg/kg VX-970, 3 mg/kg cisplatin or combination after 4 h (P-Chk1) or 48 h (P-H2AX). Extracts were prepared and immunoblotted as indicated.

Supplementary Table 1: Maximum shift in IC_{50} values for various DNA damaging agents following cotreatment with VX-970 or AZD7762 IC_{50} values for each agent were determined at 96 h by CellTiterGlo across a panel of lung cell lines. I Irinotecan, C Cisplatin, E etoposide, G gemcitabine, O Oxaliplatin

	VX-970					AZD-7762				
Cell Line	I	C	E	G	O	I	C	E	G	O
A427	9.6	12.0	10.8	4.7	3.9	3.6	4.5	3.1	5.6	1.1
A549	-1.7	2.6	-1.7	3.2	-2.0	-2.6	1.4	1.5	4.9	-1.4
A549_vector	-1.9	3.4	-1.7	2.6	-3.4	1.2	-2.4	2.1	3.4	-1.4
A549_p53	5.0	10.5	5.6	6.2	1.9	1.8	2.9	1.7	6.2	-1.1
Calu-1	16.1	2.6	131.9	4.7	1.2	7.5	2.0	6.3	4.1	NA
Calu-6	15.5	4.9	14.1	8.2	-4.1	17.6	1.4	12.0	8.2	-1.7
COR-L23	4.6	8.1	5.7	7.9	-2.0	12.9	6.0	9.9	23.8	1.6
DMS-53	1.2	4.8	1.8	2.0	-1.9	2.6	-1.5	2.6	2.3	-3.2
DMS-79	-1.2	13.0	-2.0	2.2	6.5	-1.3	-2.1	-1.8	6.3	-1.2
EBC-1	1.2	18.1	1.1	2.3	3.5	-2.9	1.5	1.2	3.6	-1.2
H1092	4.2	16.7	9.7	5.9	8.7	4.4	4.2	5.7	30.9	1.4
H1155	1.3	29.9	2.9	8.3	12.9	6.6	2.6	5.5	10.7	2.4
H1299	4.6	10.7	2.8	8.4	-1.7	3.0	3.2	1.3	13.0	1.6
H1581	8.1	16.8	7.0	9.5	5.7	6.6	3.2	2.7	12.5	-3.8
H1650	-1.3	10.8	4.4	8.6	1.3	6.2	7.7	8.3	11.5	-2.0
H1651	2.0	7.8	1.9	6.7	4.0	1.9	3.7	-3.4	6.2	2.3
H1703	-2.2	7.2	3.7	19.4	4.5	-1.9	1.8	-1.9	4.4	-2.9
H1792	9.0	14.6	3.7	2.8	2.0	3.6	4.9	3.2	7.1	1.3
H187	-2.2	3.3	-2.1	5.4	127.1	-1.5	2.1	NA	-1.3	2.2
H1993	-1.8	7.3	NA	2.8	-1.5	-3.7	3.0	NA	2.7	-2.9
H2009	3.4	10.4	2.0	9.6	-2.0	3.4	4.6	16.5	18.6	-1.6
H2087	2.6	3.9	3.2	2.4	-2.4	5.1	2.3	6.5	3.6	-2.8
H2170	1.7	24.3	2.1	4.8	3.2	-2.7	4.5	2.1	5.0	1.3
H2171	6.0	4.1	18.0	25.9	5.7	2.6	1.9	1.3	24.5	2.2
H226	1.6	2.8	-1.6	4.4	-2.2	2.8	-1.2	2.0	4.2	NA
H23	9.3	21.6	5.8	3.0	3.5	2.9	4.1	-2.5	10.6	-2.4
H460	2.0	7.9	-1.8	5.9	-1.6	-1.2	-2.9	-2.2	8.0	-1.2
H522	79.3	111.0	39.2	42.7	7.6	37.9	29.3	12.1	59.7	2.2
H661	4.4	3.7	6.2	9.9	2.2	21.1	5.9	12.7	21.8	1.6
H810	2.8	11.2	8.6	746.2	1.1	122.3	21.4	46.9	544.7	NA
H838	13.5	9.8	5.7	9.1	4.4	8.5	3.1	3.0	9.3	3.6
HCC-4006	6.6	23.1	NA	36.4	1.3	7.5	10.9	NA	53.6	2.1
HCC-827	5.0	7.2	3.0	9.2	-1.7	6.2	4.9	2.1	13.2	NA
HFL1	NA	-1.3	NA	4.1	-1.3	2.4	-1.2	NA	NA	NA
SK-MES1	-4.1	1.5	-1.6	2.9	-1.6	-2.6	-1.5	NA	1.2	NA
SW900	-1.5	3.2	-1.6	4.0	-3.0	-2.2	-1.9	1.4	3.2	-2.7

Supplementary Table 2: p53 mutational status across a panel of lung cell lines. WT: wild-type. ND: not determined.

Cell line	Tissue Type	*TP53*
A427	Adenocarcinoma	WT
A549WT	Adenocarcinoma	WT
Calu-1	Squamous cell carcinoma	WT
Calu-6	Adenocarcinoma	WT
COR-L23	Large cell carcinoma	WT
DMS-53	Small cell carcinoma	Mutant
DMS-79	Small cell carcinoma	Mutant
EBC-1	Squamous cell carcinoma	ND
H1092	Small cell carcinoma	WT
H1155	Large cell carcinoma	Mutant
H1299	Large cell carcinoma	Mutant
H1581	Large cell carcinoma	WT
H1650	Adenocarcinoma	Mutant
H1651	Adenocarcinoma	Mutant
H1703	Adenocarcinoma	Mutant
H1792	Adenocarcinoma	Mutant
H187	Small cell carcinoma	Mutant
H1993	Adenocarcinoma	Mutant
H2009	Adenocarcinoma	Mutant
H2087	Adenocarcinoma	Mutant
H2170	Squamous cell carcinoma	WT
H2171	Small cell carcinoma	Mutant
H226	Squamous cell carcinoma	WT
H23	Adenocarcinoma	Mutant
H460	Large cell carcinoma	WT
H522	Adenocarcinoma	Mutant
H661	Large cell carcinoma	Mutant
H810	Large cell carcinoma	WT
H838	Adenocarcinoma	WT
HCC-4006	Adenocarcinoma	ND
HCC-827	Adenocarcinoma	ND
HFL1	Normal	WT
SK-MES1	Squamous cell carcinoma	Mutant
SW900	Squamous cell carcinoma	Mutant

Supplementary Table 3: Summary of human primary lung tumors used in this study.

Sample ID	Gender/Age	Pathology	Grade	*TP53* Mutation
OD35982	Unknown	Squamous cell carcinoma	Poorly differentiated	R110L
OD29498	F/56	Adenocarcinoma	Poorly differentiated	Q136N
OD26749	M/66	Adenocarcinoma	Poorly differentiated	N
OD26131	M/67	Squamous cell carcinoma	Poorly differentiated	N
OD33966	Unknown	Adenocarcinoma	Moderately to poorly differentiated	N
BDG121410	M/55	Squamous cell carcinoma	Poorly differentiated	N
OD29607	M/66	Adenocarcinoma	Poorly differentiated	S127Y

Supplementary Table 4: Maximum body weight loss from studies described in Figure 4.

Tumor	Maximum Average % Body Weight Lost			
	Vehicle	VX-970	VX-970 + Cisplatin	Cisplatin
OD35982	Gain	-1.7	-6.5	-6.2
OD29498	-11.2	-6.5	-11.6	-4.8
OD26749	Gain	Gain	-8.2	-0.5
OD26131	Gain	Gain	-5.4	Gain
OD33966	-3.5	-4.1	-8.5	-6.0
BDG121410	Gain	-4.8	-4.4	-5.9
OD29607	-1.2	-2.5	-3.5	-2.6
